# Saikosaponin D suppresses enterovirus A71 infection by inhibiting autophagy

**DOI:** 10.1038/s41392-019-0037-x

**Published:** 2019-02-22

**Authors:** Chang Li, Lihong Huang, Wei Sun, Ying Chen, Ming-Liang He, Jianbo Yue, Heather Ballard

**Affiliations:** 1 School of Biomedical Sciences, University of Hong Kong, Hong Kong, China; 2 City University of Hong Kong ShenZhen Research Institute, ShenZhen, China; 3 Department of Biomedical Sciences, City University of Hong Kong, Hong Kong, China

**Keywords:** Drug screening, Cell biology

## Abstract

The dysregulation of autophagy, an evolutionarily conserved lysosomal degradation process, has been implicated in a wide variety of human diseases, and thus, small chemicals that modulate autophagy have therapeutic potential. Here, we assessed the ability of active components isolated from *Bupleurum falcatum*, a popular Chinese herb, to modulate autophagy. We found that saikosaponin D (SsD) and A (SsA) but not C (SsC) potently and reversibly inhibited the fusion of autophagosomes and lysosomes, resulting in the accumulation of autophagosomes, an increased lysosomal pH, and TFEB nuclear translocation. RAB5A knockdown or the expression of a dominant-negative RAB5 mutant significantly reduced the ability of SsD or SsA to block autophagy. Enterovirus A71 (EV-A71), the cause of hand-foot-mouth disease, has been shown to induce autophagy. We found that SsD potently inhibited EV-A71 RNA replication and subsequent viral protein synthesis, thereby preventing EV-A71-induced cell death. ATG5 knockdown inhibited EV-A71 viral protein synthesis, whereas autophagy induction by rapamycin promoted synthesis. Taken together, our data indicate that SsD and SsA are potent late-stage autophagy inhibitors that can be used to prevent EV-A71 infection.

## Autophagy: Chinese liver tonic could inhibit viral infection

Components of a popular Chinese herb known as Chaihu may inhibit the ability of the virus that causes hand-foot-and-mouth disease (HFMD) to infect cells by interfering with the natural process cells use to dispose of dysfunctional and unwanted components. Dysregulated autophagy has been implicated in various human diseases, included viral infections. Previous studies have suggested that Enterovirus A71 (EV-71), which causes HFMD, might subvert this process to aid its replication. Saikosaponins are active components of the herb Bupleurum falcatum, which appear to modulate autophagy. Jianbo Yue at the City University of Hong Kong and colleagues discovered that two such molecules, saikosaponin A and D, inhibited a key step in the autophagy process: the fusion of autophagosomes and lysosomes. This inhibited EV-A71 protein synthesis and the death of infected cells.

## Introduction

Autophagy is an evolutionarily conserved lysosomal degradation process that is essential for maintaining the homeostasis of eukaryotic cells.^1–3^ Among the three types of autophagy—macroautophagy, microautophagy, and chaperone-mediated autophagy—the most common and best-studied type is macroautophagy, which is hereafter referred to as “autophagy”. Autophagy is initiated when cytoplasmic components, e.g. damaged organelles or misfolded proteins, are sequestered by an isolated membrane called a phagophore. After cargo is sequestered by elongating this membrane, a double-membrane vesicle called an autophagosome forms. Then, a fusion process occurs between the autophagosome and a lysosome, thereby forming an autolysosome in which the sequestered components are degraded by acidic lysosomal hydrolases. Ultimately, the digested products, such as amino acids or fatty acids, are recycled back to the cytosol to maintain cell homeostasis.^4^

Autophagy involvement has been shown in a wide variety of cellular processes, including cell survival, development, proliferation, and differentiation, and the dysregulation of autophagy has been implicated in various human diseases, such as cancer, neurodegeneration, and viral infection.^5–8^ A large number of small chemicals have been found to modulate autophagy activity, and a few have emerged as potential therapeutic drugs against autophagy-related diseases.^9–11^ For example, chloroquine and hydroxychloroquine have been used alone or in combination with other chemotherapy drugs in numerous clinical or preclinical trials against cancer.^12^ However, the majority of available autophagy drugs, including chloroquine and hydroxychloroquine, are non-specific, weakly acting, or both.^13,14^ Therefore, efforts to identify specific and potent autophagy modulators are necessary to identify suitable therapeutics against autophagy-related diseases.

*Bupleurum falcatum* is a popular Chinese herb that is commonly prescribed to treat inflammatory and infectious diseases_._^15,16^ Triterpenoid saponins, including saikosaponin A (SsA), B (SsB), C (SsC), and D (SsD), are active components isolated from *Bupleurum falcatum*.^17^ For example, SsD has been shown to inhibit activated T lymphocyte proliferation by downregulating the NF*κ*B, NF-AT, and AP-1 signaling pathways_._^18^ SsD also potently inhibits the proliferation and invasion of cancer cells, induces the apoptosis of cancer cells, and suppresses angiogenesis while exhibiting low cytotoxicity to normal human lung fibroblasts_._^19^ In addition to the immunoregulatory and anticancer activity of saikosaponins, SsD, SsA, and SsB have exhibited antibacterial and antiviral effects_;_^20^ however, the mechanisms underlying antiviral effects remain unknown.

Enterovirus A71 (EV-A71), a positive single-stranded RNA virus, is in the Picornaviridae family. EV-A71 infection can cause diarrhea, rashes, and hand, foot and mouth disease (HFMD), with the potential development of severe neurological disease, especially among children.^21^ EV-A71-associated severe central nervous system disorders include polio-like acute flaccid paralysis, neurogenic pulmonary edema, and acute fatal encephalitis.^22–26^ However, there is currently no clinical drug against EV-A71 infection, and HFMD treatment is mostly restricted to antipyretic drugs, intravenous non-immune immunoglobulin, and glucocorticoids.^27^ Although inhibitors of proteases 2A^pro^, 3C^pro^, and 3D polymerase are candidate drugs for EV-A71 infection treatment,^28^ no data have been provided for these treatments in preclinical trials on non-human primate models. Interestingly, it has been reported that EV-A71 infection markedly induced autophagy, and the induced autophagy might benefit EV-A71 replication in host cells.^29–33^ Therefore, manipulating autophagic activity might provide an alternative therapeutic strategy against EV-A71 infections. Here, we assessed the ability of the active compounds SsA, SsC, and SsD isolated from *Bupleurum falcatum* to modulate autophagy and EV-A71 infection.

## Materials and methods

### Cell culture

HeLa and HEK293T cells were maintained in DMEM (Invitrogen, 12800-017) with 10% FBS (Hyclone, SH30071.01) and 100 U/ml penicillin/streptomycin (Invitrogen, 15140-122) in 5% CO_2_ at 37 °C. The cells were passaged every 2 days.

### Enterovirus-71 propagation and infection

EV-A71 (SHZH98 strain; GenBank accession number AF302996.1)^34–36^ was propagated on 90–95% confluent HeLa cells in DMEM with 10% FBS. After infection with EV-A71 for 72 h or when approximately 90% of the cells presented cytopathic effects, cells and media were collected. The collected samples underwent freeze-thaw cycles in liquid nitrogen. The lysates were centrifuged at 13,000 rpm at 4 °C for 30 min to harvest the supernatant. EV-A71 virus was stored at −80 °C until use. The viral titer was detected by median tissue culture infective dose (TCID50) using the end-point dilution assay. HeLa cells were infected with EV-A71 at a multiplicity of infection (MOI) of 1_._

### Antibodies and reagents

The following antibodies were used: LC3 (Novus, NB100–2220), SQSTM1 (MBL, PM045), EGFR (Santa Cruz, SC-03), RAB5A (Cell Signaling Technology, 3547), LAMP1 (Cell Signaling Technology, 9091), TGN46 (BIORAD AHP500GT), EV-A71 (EMD Millipore, MAB979), GAPDH (EMD Millipore, MAB374), and dsRNA (English and Scientific Consulting, J2).

The following reagents were used: SsA (HKUST RDC, 10030), SsD (HKUST RDC, 10031), SsC (HKUST RDC, 10032), glycyl-L-phenyl-alanine-ß-naphthylamide (GPN, Santa Cruz, SC-252858), thapsigargin (TG, Sigma-Aldrich, T9033), bafilomycin A1 (BAF, Sigma-Aldrich, B1793), rapamycin (Sigma-Aldrich, R8781), Fura-2 AM (Invitrogen, F1221), LysoSensor Green DND-189 (Invitrogen, L7535), LysoSensor Yellow/Blue DND-160 (Invitrogen, L7545), RNAscope® Probe-V-EV-A71-PP (Advanced Cell Diagnostics, 489071), RNAscope® Multiplex Fluorescent Detection Kit (Advanced Cell Diagnostics, 320850), TFEB siRNA (L-050607-02-0005), and nontarget siRNA (Dharmacon).

### Construction of shRNA expression vectors and production and infection of lentivirus

Two optimal 21-mers were selected in the human RAB5A^37^ and RAB7 genes. One 21-mer was selected in GFP as a control. To generate shRNA, the 21-mers were incorporated into the pLKO.1 vector. HEK293T cells were used to produce lentivirus. Briefly, HEK 293T cells were seeded at 4 × 10^5^ cells/well in 6-well plates, and the medium was replaced with antibiotic-free media the next day. PLKO.1-shRNA or pLenti-CMV-DEST vectors were mixed with the lentivirus envelope and package plasmids pMD2.G (Addgene) and psPAX2 (Addgene) in Opti-MEM for the target plasmid mixture. Another mixture of lipofectamine 2000 in Opti-MEM was prepared. After incubation for 5 min at room temperature, these two mixtures were combined and incubated for another 30 min. This mixture was added to the HEK 293 T cells. Normal media were replaced after 12 h. The viruses were harvested twice at 36 h and 60 h after transfection. For infections, cells were seeded at 2 × 10^5^ cells/well in 6-well plates. The next day, cells were infected with the targeted lentiviruses in regular medium containing 8 µg/mL polybrene. Cells were selected in fresh medium containing puromycin (3 µg/mL) 48 h after infection. After selection for 2 days, shRNA knockdown efficiencies were tested by Western blot analysis.

### Transient transfection

Cells were plated in a 24-well plate at 6 × 10^4^ cells/well. The next day, regular medium was replaced with antibiotic-free medium before transfection. Two mixtures were prepared, one containing 0.5 µl Lipofectamine 2000 in 25 µl Opti-MEM for each well, and another containing 0.5 µg DNA plasmids in 25 µl Opti-MEM/well. After 5 min incubation at room temperature, these two mixtures were combined and incubated for another 30 min. Finally, cells were transfected with the combined 50-µl mixture. The medium was replaced with fresh medium 4–6 h after transfection. After transfection for 24–48 h, the cells were ready for the following experiments.

### Western blot analyses

Cells were washed with 1x phosphate-buffered saline (PBS) twice and then lysed with ice-cold EBC protein lysis buffer. The lysates were homogenized several times by 25-gauge needles and then centrifuged at 13,200 rpm for 15 min at 4 °C to remove debris. Protein concentrations were detected by the Bradford protein assay (Bio-RAD). After denaturation by 5x SDS sample loading buffer at 99 °C for 8 min, 30–50 µg protein per sample was loaded in 8–15% SDS-PAGE gels according to the different molecular weights of the proteins. After electrophoresis, proteins were transferred to PVDF membrane (Millipore), and then the membranes were blocked with 5% non-fat milk in TBST (20 mM Tris-HCl, pH 7.6, 150 mM NaCl, 0.1% Tween-20) for 1 h at room temperature. The membrane was incubated with the appropriate primary antibodies overnight at 4 °C. The next day, the membrane was incubated with the respective secondary antibody for 1 h. Protein levels were detected by chemiluminescence after washing membranes several times and were normalized to GAPDH as a loading marker.

### Immunofluorescence analysis

After drug treatment or EV-A71 infection, cells were fixed with 4% paraformaldehyde (PFA) for 30 min at room temperature. Then, cells were blocked with blocking buffer (1% bovine serum albumin (BSA), 1% donkey serum) in PBST (1x PBS, 0.01% Tween-20 and 0.1% Triton) for 1 h and incubated with different primary antibodies overnight at 4 °C. The next day, cells were incubated with the respective secondary fluorescent antibody for 1.5–3 h and mounted with Prolong Gold Antifade Reagent (Invitrogen). Images were taken using a Carl Zeiss LSM 710 confocal microscope with a 63 × oil objective (NA 1.4) and analyzed with Zen 2011 software. The co-localization coefficient analysis of images was performed using the Zeiss LSM 710 Software as follows: (a) correct background settings for the images, (b) crop region of interest (ROI), (c) generate histogram of ROI, and (d) analyze the results, e.g., co-localized area, the ratio of co-localization, and co-localization coefficient.

### Transmission electron microscopy (TEM)

After drug treatment, the Thermanox tissue culture cover slip (with cells face up) was placed into a glass vial containing primary fixative (2.5% glutaraldehyde and 2% paraformaldehyde in 0.1 M cacodylate buffer (pH 7.2) with 0.05% CaCl_2_) for 2 h at room temperature. After rinsing the cells with 0.1 M cacodylate buffer three times for 5 min each, the cells were washed twice with 0.1 M cacodylate buffer for 10 min. Cells were then fixed with 2% OsO_4_ in 0.1 M cacodylate buffer (pH 7.4) with 1.5% potassium ferrocyanide in the dark for 2 h and were rinsed again. The cells were sequentially washed with 0.05 M cacodylate buffer, 0.025 M cacodylate buffer, and ddH_2_O for 10 min each. To dehydrate the cells, 30% ethanol, 50% ethanol, 70% ethanol, 80% ethanol, 90% ethanol, 95% ethanol, 100% ethanol, 3:1 100% ethanol:100% acetone, 1:1 100% ethanol:100% acetone, 1:3 100% ethanol:100% acetone, and 100% acetone were used in order. Subsequently, cells were infiltrated using 3:1 100% acetone:Spurr’s resin, 1:1 100% acetone:Spurr’s resin, 1:3 100% acetone:Spurr’s resin, and Pure Spurr’s resin with rotation. Next, cells were embedded in plastic mold for half a day at room temperature and then baked in an oven at 70 °C for 2 days. Ultrathin sections (approximately 90 nm) were cut and stained with 8% uranylacetate and lead citrate prior to image collection under TEM.

### MTT cell viability assay

Cells were cultured in 96-well plates at 1 × 10^4^ cells/well. After pretreatment with SsD and EV-A71 infection for 2–4 days, cells were incubated with 10% (v/v) MTT solution (USB Corporation, 19265) in regular medium for 3 h before removing solution. Immediately, 150 µL of DMSO solution was added to each well, and the absorbance of the resulting purple formazan solution was measured at 570 nm with a reference wavelength of 630 nm on a microplate reader (Techan infinite M200).

### Measurement of lysosomal pH

Cells were incubated with 1 μM LysoSensor Green DND-189, a fluorescent probe for qualitatively detecting the pH of acidic organelles, in fresh medium for 20 min at 37 °C. Fluorescence was analyzed immediately using a microplate reader with excitation and emission set at 485 and 530 nm, respectively.

In addition, a pH calibration curve was generated according to a previous protocol.^38^ Briefly, cells were plated in a 96-well plate. The next day, cells were incubated with Lysosensor Yellow/Blue DND-160 (2 μM), a ratiometric lysosomal pH dye for quantitatively determining the lysosomal pH, for 30 min at 37 °C in fresh medium, and cells were then treated with 10 μM monensin (Sigma, M5273) and 10 M nigericin (Sigma, N7143) in 25 mM 2-(N-morpholino) ethanesulfonic acid (MES) calibration buffer (5 mM NaCl, 115 mM KCl and 1.2 mM MgSO_4_, pH 3.5–6.0) for 10 min. Fluorescence was detected in a microplate reader (excitation/emission = 340/440 and 384/540 nm). The ratio of fluorescence absorbance with 340 and 380 nm excitation was plotted against the pH values in MES buffer using Microsoft Excel software.

### RNA in situ hybridization

To visualize the EV-A71 genome, in situ RNA hybridization was performed.^39^ Cells were seeded on cover glasses in 48-well plates at 3 × 10^4^ cells/well. After drug treatments and EV-A71 infection, cells were fixed with 10% neutral buffered formalin (NBF) for 30 min at room temperature. The cells were washed with 1x PBS twice, and then diluted protease (1:15) was added to cells with 1× PBS to release RNA in the cells. After incubation with diluted protease for 10 min, cells were hybridized with the target probe for 2 h at 40 °C and then sequentially with Amp 1-FL (30 min), Amp 2-FL (15 min), Amp 3-FL (30 min), and Amp 4-FL (15 min) at 40 °C to amplify the RNA signal. Before each amplification process, the cells were washed twice with 1× wash buffer. After incubation with blocking buffer for 1 h, experiments were performed as described above in the immunofluorescence analysis section.

### Quantification of intracellular and extracellular viral genomic RNA

HeLa cells were pretreated with SsD for 4 h, washed twice with 1× PBS, and infected with EV-A71 at an MOI of 1. EV-A71 virus was allowed to be internalized into cells for 1 h. Unattached viruses were washed out twice with PBS, and 200 μl regular medium containing 2% FBS was added to each well. Total RNA was isolated from infected cells or culture medium to measure the intracellular viral RNA or extracellular viral RNA in the virions at different time points, and qRT-PCR assays were performed.^34^

### Virus titration assay

HeLa cells were pretreated with SsD for 4 h and then washed twice with 1× PBS. Ten-fold dilutions of EV-A71 stock were prepared before EV-A71 infection. EV-A71 virus was allowed to be internalized into cells for 1 h. After incubation for 1 h, cells were covered with agar, which formed a gel. A circular zone-like plaque was formed by each infectious particle. To stain living cells and distinguish between living cells and plaques, crystal violet was added to cells. The titer of EV-A71 was quantified as plaque-forming units (PFU) per milliliter and calculated by counting plaques. Each dilution was plated in triplicate to ensure accuracy.

### Intracellular Ca^2+^ measurement

Cells were seeded at 7 × 10^4^ cells per well in a 24-well plate. The next day, cells were pretreated with SsD for 6 h and then incubated with Fura-2 AM (4 µM) in regular HBSS (Invitrogen, 14175-059) at room temperature for 20–30 min in the dark. Subsequently, cells were washed three times with calcium-free HBSS containing 2 mM EGTA (Sigma, 3889). Finally, cells were placed on the stage of an Olympus inverted epifluorescence microscope with a ×20 objective and challenged with 200 µM GPN or 1 µM TG at room temperature for 10 min. Fluorescence images were collected by excitation alternating between 340 nm and 380 nm with emission at 510 nm. Images were captured every 5 s and processed by *Cell R* imaging software.

### Statistical analysis

Data are presented as the means ± S.E.M. The statistical significance of differences was determined by one-way ANOVA or Student’s *t* test. *P* < 0.05 was regarded as significant.

## Results

### *SsD* and *SsA* inhibit autophagosome-lysosome fusion in HeLa cells

**Fig. 1:**
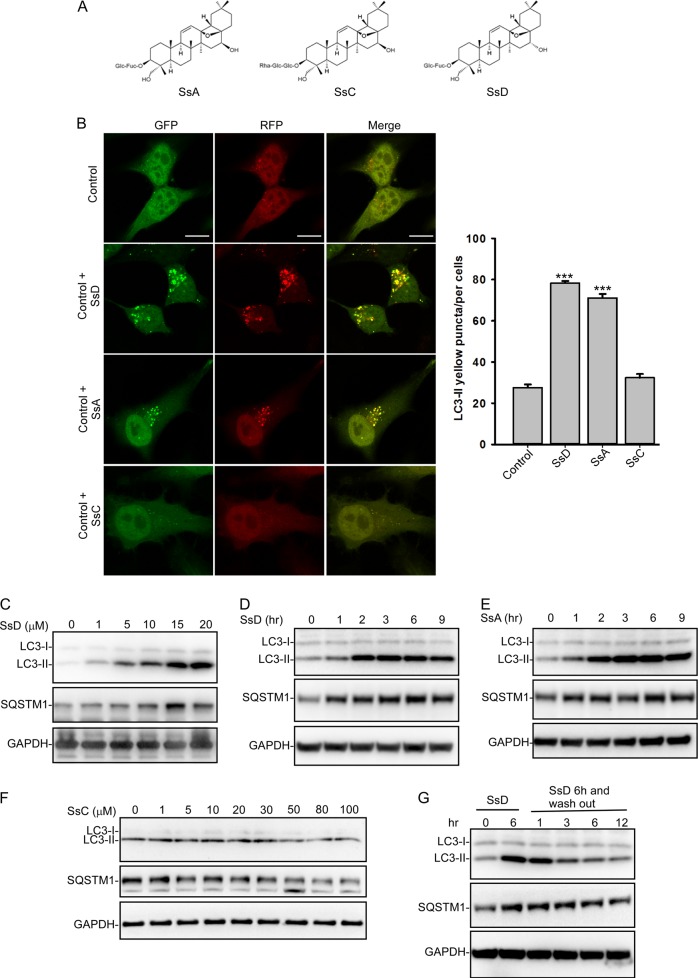
SsD and SsA inhibit autophagy in HeLa cells. **a** Structures of SsA, SsC, and SsD. **b** SsD (15 μM) and SsA (30 μM), but not SsC, significantly increased LC3-II yellow puncta formation but did not significantly affect LC3-II red-only puncta in RFP-GFP-LC3-expressing HeLa cells. Scale bar = 10 μm. Quantification of LC3 yellow puncta/red puncta (%) is presented as the mean ± S.E., *n* = ~80 cells from 3 independent experiments. **c** Treatment of HeLa cells with SsD for 6 h induced the accumulation of both LC3-II and SQSTM1 in a dose-dependent manner. **d** SsD (15 μM) induced the accumulation of both LC3-II and SQSTM1 in HeLa cells in a time-dependent manner. **e** SsA (30 μM) induced the accumulation of both LC3-II and SQSTM1 in HeLa cells in a time-dependent manner. **f** Treatment with different concentrations of SsC for 6 h failed to induce the accumulation of either LC3-II or SQSTM1 in HeLa cells. **g** SsD (15 μM) reversibly inhibited autophagy

**Fig. 2:**
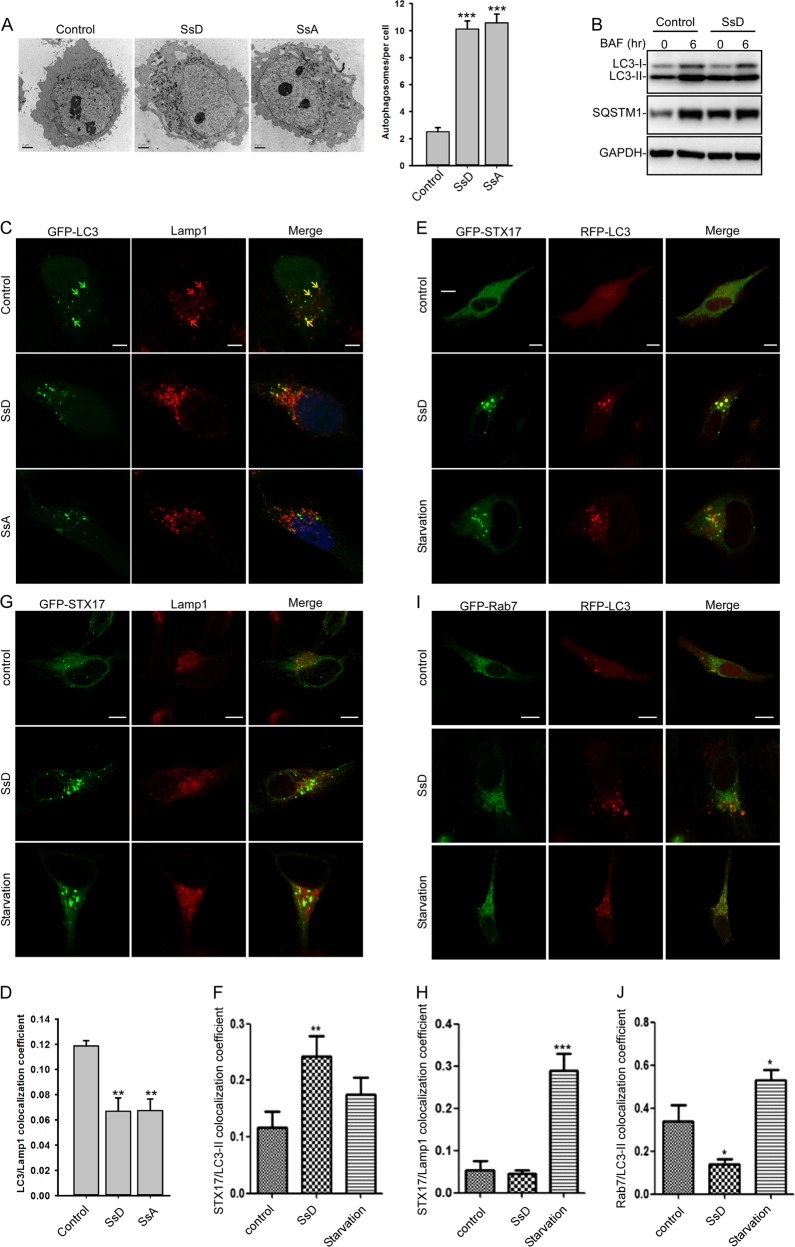
SsD induces the accumulation of autophagosomes. **a** SsD (15 μM) and SsA (30 μM) induced the accumulation of autophagic vacuoles as shown in the electron micrographs. Quantification of autophagosomes per cell is presented as the mean ± S.E., *n* = ~20 to 40 cells. The asterisks (*) symbols indicate *p* < 0.05 by *t* test analysis. **b** BAF (100 nM) treatment for 6 h failed to further induce the accumulation of either LC3-II or SQSTM1 in control or SsD (15 μM)-treated HeLa cells. **c**, **d** HeLa cells were transfected with GFP-LC3 and treated with or without SsD (15 μM) or SsA (30 μM) before Lamp1 immunostaining and confocal imaging (**c**). The quantification of LC3-II/Lamp1 co-localization is presented as the mean ± S.E., *n* = ~80 to 100 cells of 3 independent experiments (**d**). **e**, **f** HeLa cells were transfected with GFP-STX17 and RFP-LC3 and treated with or without SsD (15 μM) or underwent starvation followed by confocal imaging (**e**). The quantification of LC3-II/STX17 co-localization is expressed as the mean ± S.E., *n* = 3 (totally 15–30 cells) (**f**). **g**, **h** HeLa cells were transfected with GFP-STX17 and treated with or without SsD (15 μM) or underwent starvation prior to Lamp1 immunostaining and confocal imaging (**g**). The quantification of STX17/Lamp1 co-localization is expressed as the mean ± S.E., n = 3 (totally 15–30 cells) (**h**). **i**, **j** HeLa cells were transfected with GFP-RAB7 and RFP-LC3 and treated with or without SsD (15 μM) or underwent starvation prior to confocal imaging (**i**). The quantification of RAB7/LC3-II co-localization is expressed as the mean ± S.E., *n* = 3 (totally 15–30 cells) (**j**). Scale bar = 10 μm. Differences between control group and treatment groups were analyzed by one-way ANOVA

Triterpenoid saponins, e.g., SsA, SsC, and SsD, are the active compounds in *Bupleurum falcatum* (Fig. 1a). We first assessed the ability of these three saponins to modulate autophagy in RFP and GFP tandem-tagged LC3-expressing HeLa cells. As shown in Fig. 1b, both SsD and SsA, but not SsC, markedly induced yellow LC3-II puncta formation in HeLa cells, whereas almost no red-only LC3-II puncta were present in SsD-treated or SsA-treated cells. These data suggested that the fusion between autophagosomes and lysosomes is blocked in SsD-treated or SsA-treated cells. Likewise, the treatment of cells with SsD or SsA, but not SsC, markedly increased the levels of both LC3-II and p62, a cargo receptor for the autophagic degradation of ubiquitinated proteins, in both time-dependent and concentration-dependent manners (Fig. 1c–f), which also suggested the inhibition of autophagosomal-lysosomal fusion in SsA-treated or SsD-treated cells. Interestingly, we also found that the accumulated LC3-II and p62 (SQSTM1) in SsD-treated cells was reduced after washing out SsD, indicating that the effects of SsD on autophagy are reversible (Fig. 1g). We further obtained transmission electron microscope images of HeLa cells treated with SsD or SsA. As shown in Fig. 2a, SsD or SsA treatment of cells significantly increased the number of autophagosome-like structures compared to control cells. Because SsD and SsA are epimers with very similar effects on autophagy, we primarily studied SsD in the subsequent studies.

To confirm that SsD inhibits the fusion between autophagosomes and lysosomes, we treated control or SsD pre-treated HeLa cells with bafilomycin A1 (BAF), a vacuolar proton ATPase inhibitor that can arrest autophagosomal-lysosomal fusion in cells. As shown in Fig. 2b, BAF treatment did not induce further accumulation of LC3-II and p62 in SsD-treated HeLa cells compared to control cells. Similar results were observed in SsA-treated cells (Fig. S1). These data suggested that SsD and SsA, like BAF, inhibit autophagosome-lysosome fusion, thereby resulting in the accumulation of both LC3-II and p62. To further confirm that the SsD-induced LC3-II puncta represent autophagosomes, we assessed the co-localization of LC3-II, Lamp1 (a lysosomal marker), STX17 (a SNARE protein locating at the autophagosome), and RAB7 (a small GTPase located at late endosome or lysosome) in SsD-treated cells. SsD-induced or SsA-induced LC3-II puncta exhibited weak co-localization with Lamp1 (Fig. 2c, d). However, SsD-induced LC3-II puncta were almost completely co-localized with STX17 puncta (Fig. 2e, f). Likewise, SsD-induced STX17 puncta exhibited weak co-localization with Lamp1, whereas starvation significantly induced their co-localization (Fig. 2g, h). Moreover, SsD failed to induce the co-localization between LC3-II and RAB7, whereas starvation significantly induced co-localization (Fig. 2d). Taken together, these data demonstrated that the SsD-induced LC3-II puncta represented autophagosomes, not autolysosomes. Therefore, our results indicated that SsD and SsA, like BAF, inhibit the fusion between autophagosomes and lysosomes, resulting in the accumulation of autophagosomes and increased levels of LC3-II and p62.

### SsD alkalizes lysosomal pH and induces TFEB nuclear translocation in HeLa cells

**Fig. 3:**
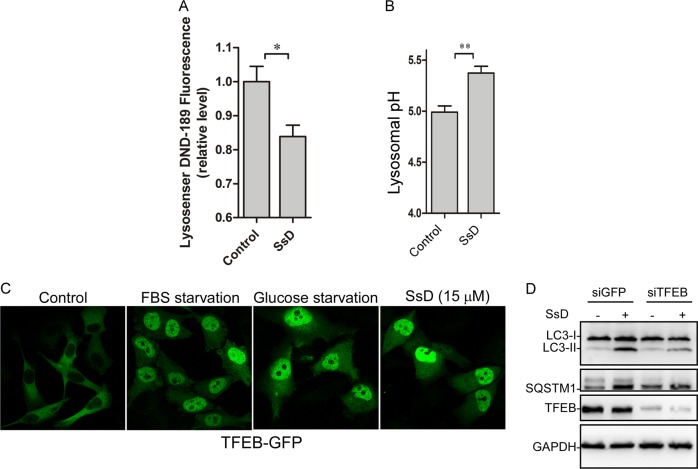
SsD treatment of HeLa cells increases lysosomal pH and induces TFEB nuclear translocation. **a**, **b** SsD (15 μM) led to an increase of lysosomal pH in HeLa cells as measured using Lysosensor DND-189 (**a**) or the quantitative ratiometric LysoSensor Yellow/Blue DND-160 (**b**). The graphs in (**b**) and (**c**) represent data from three independent experiments, and data are expressed as the means ± S.D., *n* = 3. The asterisk (*) symbols indicates *p* < 0.05 by *t*-test analysis. **c** HeLa cells were transfected with TFEB-GFP and treated with or without SsD (15 μM) or underwent starvation before confocal imaging. **d** HeLa cells were transfected with siGFP or siTFEB and treated with or without SsD (15 μM), and then LC3, p62, TFEB, or GAPDH immunoblot analyses were performed

Because acidic lysosomal pH is essential for autophagosome-lysosome fusion,^40^ we next examined whether SsD treatment of cells changes lysosomal pH. Cells were labeled with LysoSensor Green DND-189 (pKa = ~5.2);^41^ its fluorescence intensity is decreased when pH is increased. As shown in Fig. 3a, SsD treatment significantly decreased the fluorescence intensity of lysosomes, suggesting that lysosomal pH is increased in SsD-treated cells. Furthermore, lysosomal pH was quantified by the quantitative ratiometric LysoSensor Yellow/Blue DND-160^42^ assay. As shown in Fig. 3b, lysosomal pH was increased from pH 4.99 in control cells to pH 5.37 in SsD-treated cells. Similar results were observed in SsA-treated cells (Fig. S2). Therefore, these data indicated that SsD and SsA, again with similarities to BAF and chloroquine, alkalizes lysosomal pH, which likely contributes to the autophagosome-lysosome fusion blockage.

It has been shown previously that TFEB, a transcription factor important for autophagy progression, is associated with lysosomes when cells are maintained in normal medium due to its phosphorylation by mTOR, whereas starvation triggers the nuclear localization of dephosphorylated TFEB because of the inactivation of mTOR.^43,44^ We therefore examined whether SsD treatment alters TFEB lysosomal localization. As shown in Fig. 3c and S3A, SsD treatment, similarly to FBS or glucose starvation, markedly induced the nuclear localization of TFEB. However, SsD treatment did not affect mTOR activation (Fig. S3B), suggesting that SsD-induced TFEB nuclear localization is mTOR independent. As expected, TFEB knockdown abolished SsD-induced LC3-II and p62 accumulation (Fig. 3d), which is in agreement with the general role of TFEB in autophagy progression and lysosome biogenesis.^44,45^

### SsD delays the endocytosis pathway in HeLa cells and does not alter Ca^2+^ content in lysosomes or the ER

**Fig. 4:**
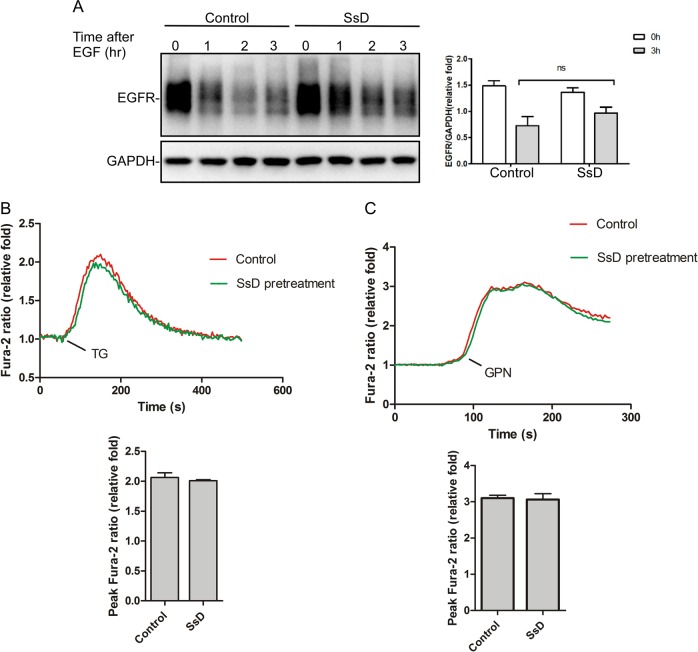
SsD delays the endocytosis pathway in HeLa cells and does not alter Ca^2+^ content in the lysosome or ER. **a** HeLa cells were treated with EGF in the presence or absence of SsD (15 μM) and subjected to EGFR immunoblot analyses. **b** SsD (15 μM) pretreatment for 6 h failed to affect the ability of TG (1 µM) to release ER Ca^2+^ in Fura-2-loaded HeLa cells in the absence of extracellular Ca^2+^. **c** SsD (15 μM) pretreatment for 6 h did not affect the ability of GPN (200 µM) to release lysosomal Ca^2+^ in Fura-2-loaded HeLa cells in the absence of extracellular Ca^2+^. The graphs in (**b**) and (**c**) represent data from three independent experiments, and data are expressed as the means ± S.D., *n* = 3. The * symbols indicate *p* < 0.05 by *t*-test analysis

Because the lysosomal pH was increased in SsD-treated and SsA-treated cells, we performed EGFR (epidermal growth factor receptor) degradation assays to assess whether SsD affects the general endosomal-lysosomal pathway. HeLa cells were pre-treated with or without SsD before EGF addition. EGF binding to its receptor (EGFR) triggered EGFR translocation from the plasma membrane to the lysosome to be degraded; however, SsD pretreatment only delayed but did not block the degradation of EGFR (Fig. 4a), suggesting that SsD has marginal effects on general endosomal-lysosomal degradation.

Because intracellular Ca^2+^ is important for autophagy progression and membrane fusion^46–49^ and because it was previously suggested that SsD could inhibit sarco/endoplasmic reticulum Ca^2+^ ATPase (SERCA) in the ER to trigger ER Ca^2+^ release, we assessed the effects of SsD on ER Ca^2+^ content. As shown in Fig. 4b, thapsigargin (TG), an inhibitor of SERCA, triggered increases in cytosolic Ca^2+^ to similar levels in the presence or absence of SsD pretreatment, indicating that ER Ca^2+^ pools are not affected by SsD treatment. Lysosomes are also intracellular Ca^2+^ pools, and Ca^2+^ and protons are strongly coupled to maintain acidic pH in lysosomes.^50^ Therefore, we examined the effects of SsD treatment on the lysosomal Ca^2+^ content by measuring the cytosolic Ca^2+^ changes elicited by Glycyl-l-phenylalanine 2-naphthylamide (GPN), which specifically breaks the lysosomal membrane^51^ to release lysosomal Ca^2+^.^52^ As shown in Fig. 4c, GPN induced similar levels of cytosolic Ca^2+^ increases in the presence or absence of SsD pretreatment, indicating that SsD does not change lysosomal Ca^2+^ content. Taken together, these data suggest that SsD-mediated autophagy inhibition is independent of ER or lysosomal Ca^2+^ changes.

### RAB5 is required for autophagy inhibition by SsD

**Fig. 5:**
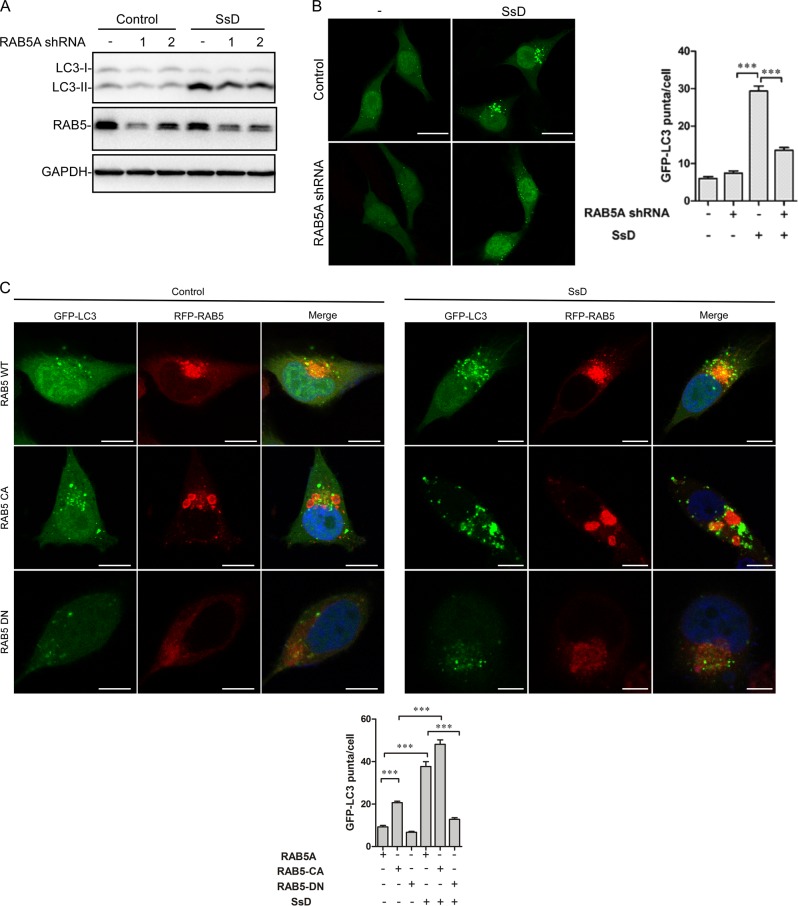
RAB5 is required for autophagy inhibition by SsD. **a** RAB5A knockdown inhibited SsD (15 μM)-induced accumulation of LC3-II in HeLa cells. **b** RAB5A knockdown inhibited SsD (15 μM)-induced accumulation of GFP-LC3-II puncta in HeLa cells. Scale bar = 10 μm. Quantification of GFP-LC3-II puncta per cell is presented as the mean ± S.E., *n* = ~50 cells of 3 independent experiments. The asterisk (*) symbols indicate *p* < 0.05 by *t* test analysis. **c** Overexpression of a constitutively active form of RAB5A (RAB5A-CA) enhanced the inhibitory effect of SsD, whereas the overexpression of a dominant-negative form of RAB5A (RAB5A-DN) prevented the increase of GFP-LC3 puncta induced by SsD. Scale bar = 10 μm. Quantification of GFP-LC3-II puncta per cell is presented as the mean ± S.E., *n* = ~50 cells of 3 independent experiments

Growing evidence indicates that RAB small GTPases play important roles in autophagy regulation,^53^ and thus, we assessed the role of RAB small GTPase in SsD-mediated autophagy inhibition. RAB7 knockdown in HeLa cells induced the accumulation of LC3-II, but SsD, SsA, or BAF treatment of RAB7-knockdown cells failed to further increase LC3-II levels, again suggesting that SsD and SsA, similarly to BAF, inhibit the autophagosomal-lysosomal fusion (Fig. S4). In contrast, the knockdown of RAB5A, a small GTPase known to be involved in early endocytosis,^54^ markedly reversed the SsD-induced accumulation of LC3-II compared to control HeLa cells (Fig. 5a). Similarly, the SsD-induced accumulation of GFP-LC3 puncta was significantly inhibited in RAB5A knockdown cells compared to control cells (Fig. 5b). Likewise, the overexpression of a dominant-negative form of RAB5A (RAB5A-DN)^55^ significantly inhibited SsD-induced GFP-LC3 puncta formation compared to control cells, whereas the overexpression of a constitutively active form of RAB5A (RAB5A-CA)^55^ enhanced the SsD-induced accumulation of LC3-II puncta (Fig. 5c). Interestingly, RAB5A-CA overexpression alone also markedly induced GFP-LC3 puncta formation in HeLa cells, suggesting defects in autophagosome-lysosome fusion (Fig. 5c). Similar results were observed in SsA-treated cells (Fig. S5A–S5C). In summary, these data clearly demonstrated that RAB5 is required for autophagy inhibition by SsD or SsA.

### SsD inhibits EV-A71 infection in HeLa cells

**Fig. 6:**
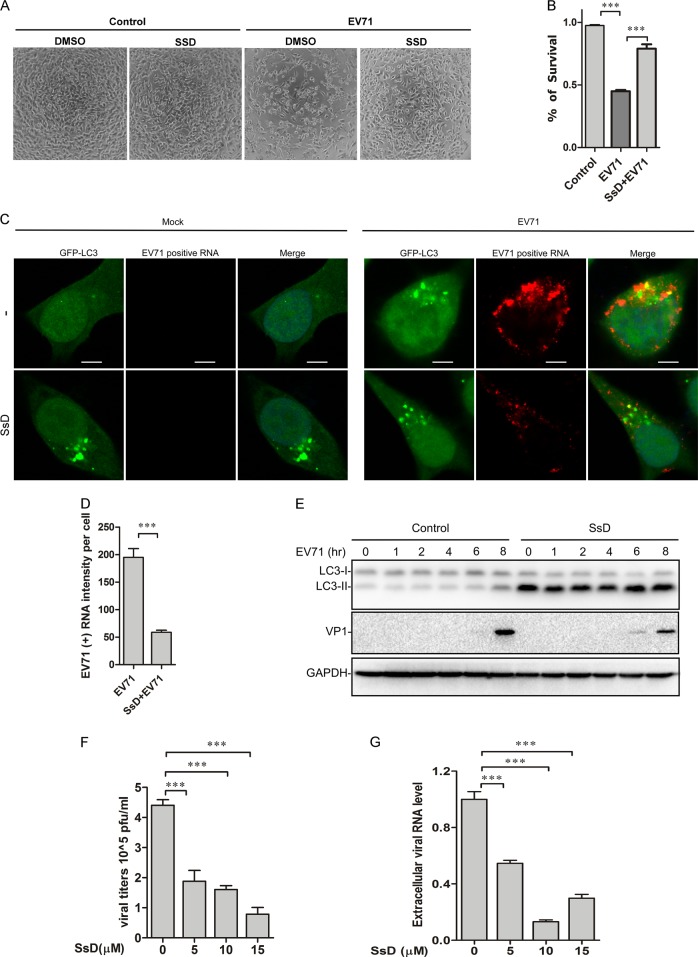
SsD inhibits EV-A71 replication in HeLa cells. **a** HeLa cells pretreated with or without SsD (15 μM) were infected with EV-A71 (MOI = 1) for 12 h. SsD pretreatment greatly reduced the number of cytopathic cells after EV-A71 infection. **b** HeLa cells were infected with EV-A71 (MOI = 1) in the presence or absence of SsD (15 μM) for 12 h, and MTT assays were performed. The graph represents data from three independent experiments, and data are expressed as the mean ± S.D., *n* = 3. The asterisk (*) symbols indicate *p* < 0.05 by *t* test analysis. **c** and **d** GFP-LC3-expressing HeLa cells were infected with EV-A71 (MOI = 1) in the presence or absence of SsD (15 μM) for 5 h, and EV-A71 positive strand RNA hybridization was performed (**c**). Quantification of EV-A71 positive RNA intensity per cell is expressed as the mean ± S.E., *n* = ~50 cells from 3 independent experiments. The asterisk (*) symbols indicate *P* < 0.05 by Student’s *t* Test analysis (**d**). **e** HeLa cells were infected with EV-A71 (MOI = 1) in the presence or absence of SsD (15 μM) for the indicated times, followed by LC3, VP1, and GAPDH immunoblot analyses. **f** Viral titers were significantly decreased by SsD treatment in a dose-dependent manner after EV-A71 infection. WT HeLa cells were infected with EV-A71 (MOI = 1) for 12 h. **g** SsD (15 μM) treatment markedly reduced extracellular EV-A71 RNA level in a dose-dependent manner. WT HeLa cells were infected with EV-A71 (MOI = 1) for 12 h

It has been shown that EV-A71 induces autophagy in host cells, which might in turn facilitate the synthesis of viral RNA and proteins.^29–33^ Therefore, we reasoned that SsD could inhibit autophagy to suppress EV-A71 infection. As expected, upon EV-A71 (MOI = 1) infection, many cells developed cytopathic effects, showing a rounded shape or even detaching from the culture well. Treatment with SsD during EV-A71 infection greatly decreased the number of cytopathic cells, whereas SsD (15 μM) alone showed little toxicity in HeLa cells (Fig. 6a). To better quantify the protective effect of SsD against EV-A71 infection, an MTT cell viability assay was performed. As expected, SsD significantly inhibited EV-A71-induced HeLa cell death (Fig. 6b).

To determine whether the anti-EV-A71 function of SsD results from its inhibition of the late stage of autophagy, we detected the EV-A71 positive RNA strand by RNA in situ hybridization. As shown in Fig. 6c, d, the EV-A71 positive RNA strand was significantly reduced by SsD treatment compared to the level in EV-A71-infected cells without SsD treatment, indicating that the replication of EV-A71 viral RNA was inhibited by SsD treatment. Likewise, the expression level of VP1 was significantly decreased by SsD treatment during EV-A71 infection (Fig. 6e). To further detect the antiviral activity of SsD, we measured the viral titers in the culture medium. As shown in Fig. 6f, viral titers were significantly decreased by treatment with SsD in a dose-dependent manner. In addition, we detected secreted EV-A71 virions in the culture medium by measuring extracellular EV-A71 RNA. Treatment with SsD markedly reduced the extracellular EV-A71 RNA level during EV-A71 infection (Fig. 6g).

**Fig. 7:**
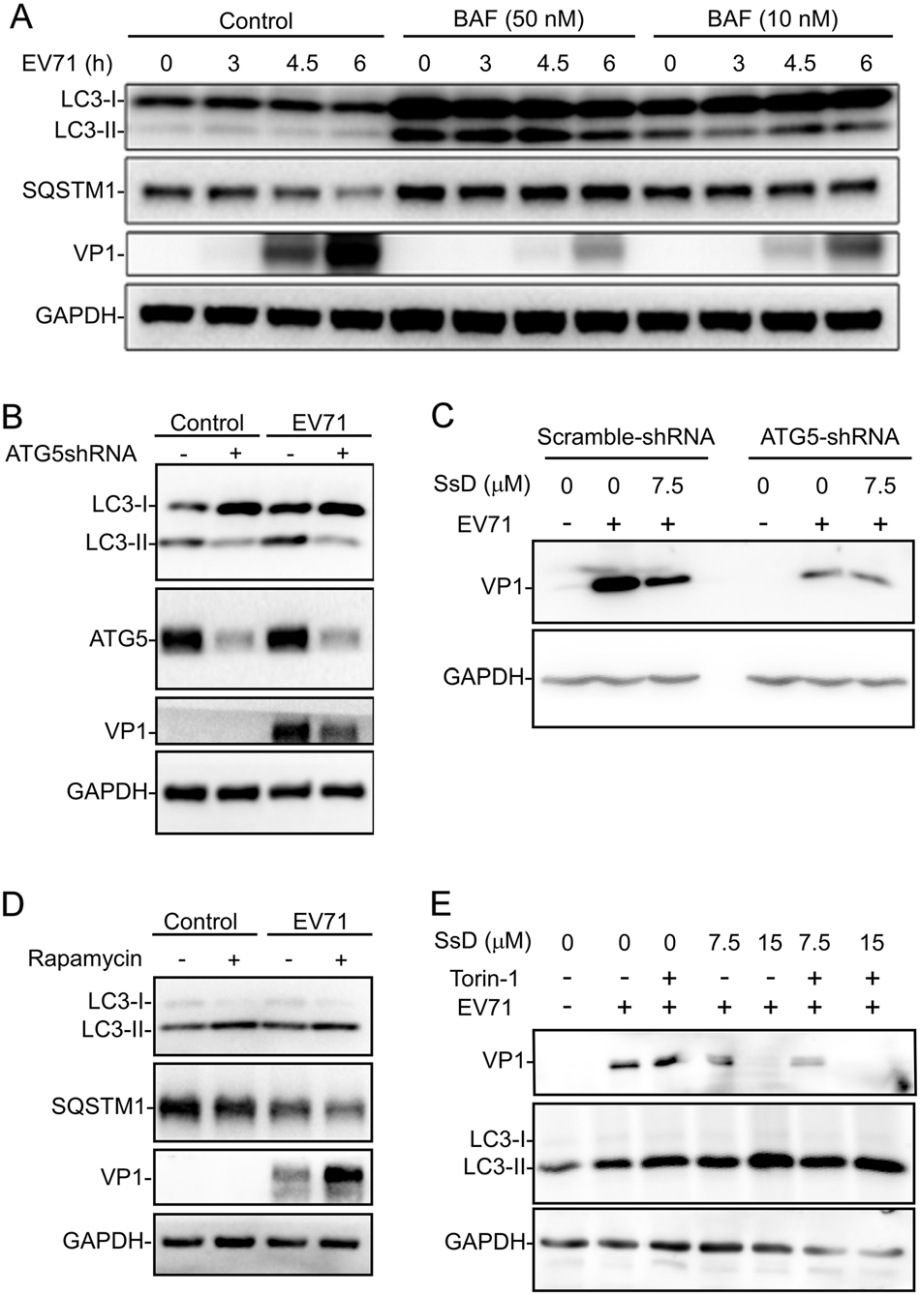
Autophagy is involved in EV-A71 replication in HeLa cells. **a** VP1 synthesis was dramatically reduced by BAF (50 or 10 nM) treatment in EV71-infected cells compared to cells without BAF treatment. **b** ATG5 knockdown markedly inhibited EV-A71 infection in HeLa cells. **c** SsD treatment failed to further inhibit AV-A71 infection in ATG5-knockdown HeLa cells. **d** Rapamycin treatment enhanced EV-A71 infection in HeLa cells. **e** SsD treatment markedly inhibited EV-A71 infection in HeLa cells treated with or without Torin-1

To further assess the role of autophagy in EV-A71 infection of cells, we infected HeLa cells with EV-A71 in the presence of BAF, a known autophagosome-lysosome fusion inhibitor similar to SsD. As expected, the treatment of HeLa cells with BAF dosedependently inhibited the expression of VP1 induced by EV-A71 infection (Fig. 7a). Likewise, the knockdown of ATG5, an important protein for early autophagy progression, markedly inhibited the expression of VP1 after EV-A71 infection in HeLa cells (Fig. 7b); however, the SsD treatment of ATG5-knockdown HeLa cells failed to further inhibit VP1 expression after EV-A71 infection (Fig. 7c). In contrast, we found that treatment of HeLa cells with rapamycin, an mTOR inhibitor known to induce autophagy, markedly increased the levels of VP1 protein (Fig. 7d). Interestingly, the SsD treatment of cells markedly inhibited VP1 levels induced by EV-A71 infection in the presence or absence of Torin-1, another mTOR inhibitor (Fig. 7e). Taken together, these data suggested that the ability of SsD to inhibit the late stage of autophagy contributes to its anti-EV-A71 effects_._

## Discussion

Here we found that the treatment of cells with SsD or SsA, active components in *Bupleurum falcatum*, inhibited autophagosome-lysosome fusion and resulted in the accumulation of autophagosomes (Figs. 1 and 2). We also found that SsD or SsA treatment increased lysosomal pH and induced the nuclear localization of TFEB (Fig. 3) but did not affect ER or lysosome Ca^2+^ pools (Fig. 4). Moreover, we found that RAB5 was required for SsD-induced or SsA-induced autophagosomal-lysosomal fusion blockage (Fig. 5), but SsD and SsA failed to further inhibit autophagosomal-lysosomal fusion in RAB7 knockdown cells (Fig. S4). Interestingly, we found that SsD strongly reduced EV-A71 RNA replication, viral protein synthesis, and virus titers in host cells (Fig. 6a–g), and this inhibition was correlated with inhibitory effects on autophagy. Likewise, autophagy inhibition by ATG5 knockdown or BAF treatment suppressed EV-A71 viral replication (Fig. 7a, b), whereas autophagy induction by rapamycin or Torin-1 promoted replication (Fig. 7d, e). Thus, we speculated that autophagy inhibition by SsD or SsA acts as a protective mechanism against EV-A71 infection.

Previous research showed that SsD treatment of cells increases LC3-II levels and LC3-II puncta, but the authors concluded that SsD induced earlier autophagy, which ultimately resulted in autophagic cell death.^56^ Here, we found that the SsD-induced LC3-II puncta failed to co-localize with LAMP1 or RAB7, two lysosomal markers (Fig. 2c, i), but exhibited strong co-localization with STX17, an autophagosomal marker (Fig. 2e). Moreover, SsD failed to further increase LC3-II levels in BAF-treated or RAB7 knockdown cells (Fig. 2b and S4). Taken together, these data clearly indicate that SsD and SsA inhibit the autophagosome-lysosome fusion, and the increased levels of LC3-II and p62 and accumulated LC3-II puncta in SsD-treated cells are due to the blockage of autophagosomal-lysosomal fusion rather than autophagy induction at earlier stages.

We also found that RAB5A was required for autophagy arrest by SsD or SsA (Fig. 5 and S5). We speculate that SsD and SsA might indirectly activate RAB5, which subsequently contributes to the defects in lysosome biogenesis and results in lysosomal pH elevation. RAB5 is a small GTPase. GTP-bound RAB5 is the active form, whereas GDP-bound RAB5 is the inactive form. A guanine nucleotide exchange factor (GEF) is responsible for the conversion from GTP-bound RAB5 to GDP-bound RAB5. Both the GTPase-activating protein (GAP) and GTPase activity of RAB5 are involved in the switch from RAB5-GTP to RAB5-GDP. Many GEFs and GAPs of RAB5 have been discovered.^57^ There are several possibilities for RAB5A activation by SsD. One is that SsD directly inhibits RAB5 GTPase activity; another is that a RAB5 GEF is activated by SsD or a RAB5 GAP is inhibited by SsD, thereby activating RAB5 as a RAB5-GTP form. Notably, we recently found that the small chemical vacuolin-1 also indirectly activates RAB5 to arrest autophagosome-lysosome fusion.^37^

To investigate the role of autophagy in EV71 infection, HeLa cells were selected in this study to undergo EV71 infection. HeLa cells are susceptible to EV-A71 infection, enabling efficient progeny viral propagation. The viral growth kinetics and the cytopathic effects of EV-A71 in HeLa cells are similar to those of other popularly used cell lines to study EV-A71. HeLa cells have been used to study enteroviruses, including EV-A71.^58–60^ In addition, HeLa cells are autophagy competent.^37,61^ However, it is worth noting that HeLa cells are less susceptible to EV-A71 infection than Rhabdomyosarcoma (RD) cells or Vero cells, two popular cell lines used to study EV71, and thus, a higher titer of EV-A71 virus was used to infect HeLa cells.

## Supplementary information

Supplemental figures
