# Supplementary figures and images for: Saikosaponin D suppresses enterovirus A71 infection by inhibiting autophagy
Source: Signal Transduct Target Ther. 2019 Feb 22;4:4. doi: 10.1038/s41392-019-0037-x (PMC6385247; doi:10.1038/s41392-019-0037-x)

Fig. S1

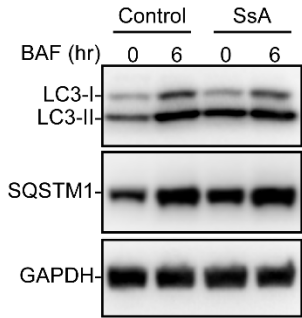

Fig. S2

A

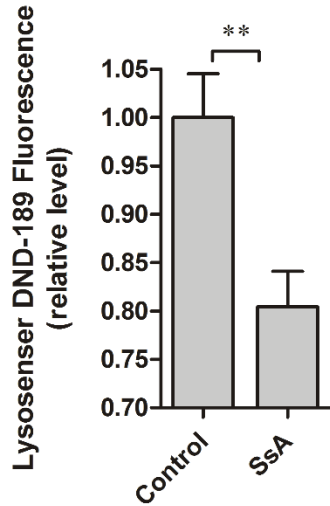

B

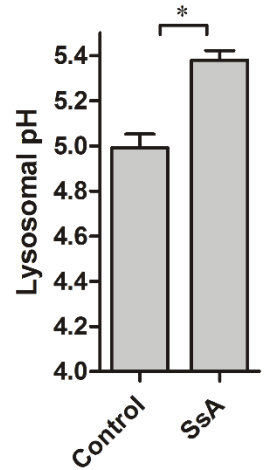

Fig. S3

A

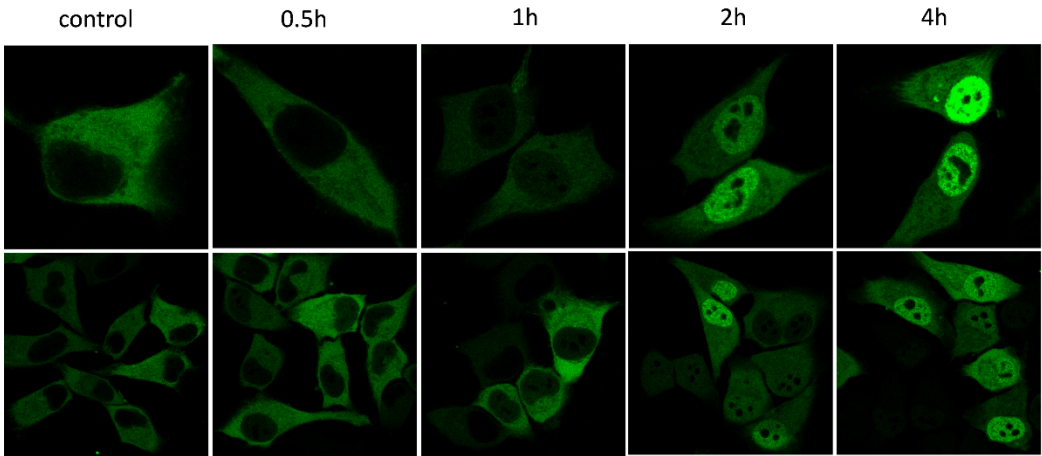

B

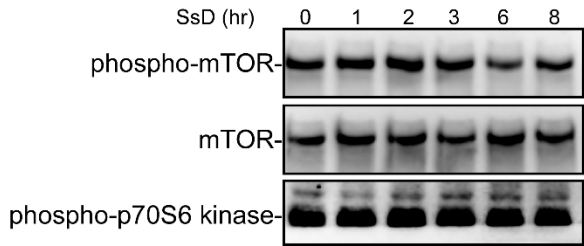

Fig. S4

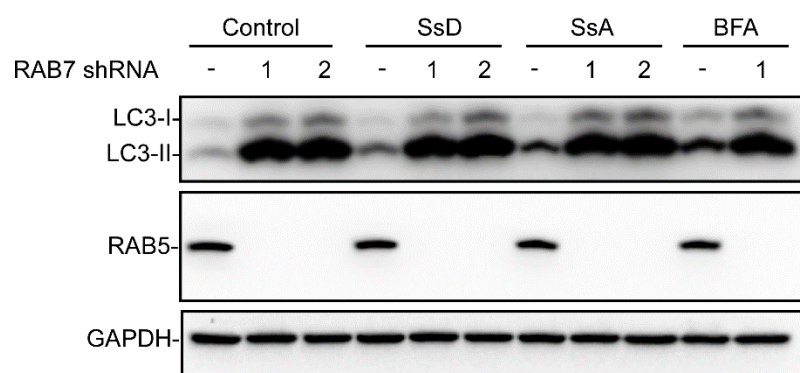

Fig. S5

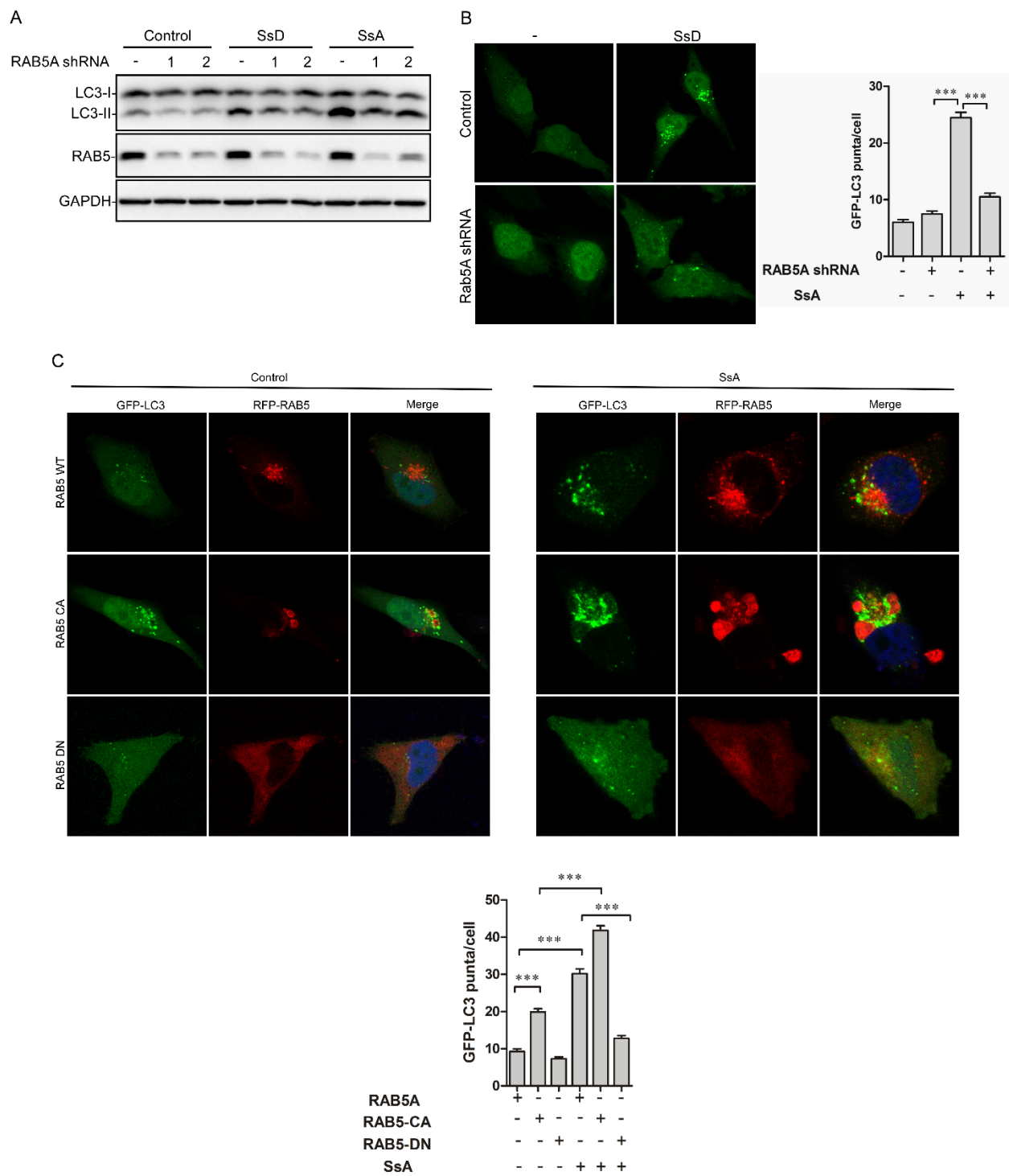

Supplement: Supplementary file 1 — Supplemental figures [file 41392_2019_37_MOESM1_ESM.pdf]
